# SG-APSIC1063: Building and application of e-learning software for infection control, Cho Ray Hospital

**DOI:** 10.1017/ash.2023.63

**Published:** 2023-03-16

**Authors:** Nguyen Xuan Nhat Duy, Nguyen Xuan Nhat Duy, Phan Thi Hong Thuy

**Affiliations:** Cho Ray Hospital, Ho Chi Minh City, Vietnam; 201b Nguyen Chi Thanh, 12 Ward, 5 District, Ho Chi Minh City, Vietnam; 201b Nguyen Chi Thanh, 12 Ward, 5 District, Ho Chi Minh City, Vietnam

## Abstract

**Objectives:** In the context of the COVID-19 pandemic over the past 2 years, training regarding infection and prevention control (IPC) has become essential in responding promptly to the pandemic. Many healthcare workers from Cho Ray Hospital and provincial hospitals need IPC training; however, human resources and facilities for continuous education and training are lacking. Therefore, IPC e-learning has become necessary for medical staff, and we designed IPC e-learning courses to meet healthcare workers’ needs for efficient, time and cost-saving training to ensure safety during the COVID-19 pandemic. **Methods:** All medical staff of Cho Ray Hospital were invited to participate in the infection control e-learning study. The software was developed based on the existing lectures from practical infection control protocols. Healthcare workers were asked to study the software and take a test on the their training. **Results:** We built the e-learning course of IPC for 5,000 participants as well as management software to manage lessons, member data, and test results. After implementation for 2 months in the hospital, 207 participants had taken the exam 2,234 times. Overall, 70.5% of participants were nurses and 14.9% were doctors. Moreover, 66.4% of participants passed the test the first time they took it, and 33.6% took the test a second time. After the second test, the percentage of members who passed the exam was 100%. **Conclusions:** Building and applying e-learning software for IPC training has brought about efficiency and quality of training, has reduced the use of human resources for training, and has decreased costs. The software application is being expanded to all hospitals in Vietnam.

